# Sex differences in children and adolescents with attention-deficit/hyperactivity disorder: a literature review

**DOI:** 10.3389/frcha.2025.1582502

**Published:** 2025-06-19

**Authors:** Danilo Dimitri, Giuliana Delia, Francesco Cavallo, Matteo Varini, Franco Fioretto

**Affiliations:** ^1^ Department of Psychology, Univeristy of Turin, Turin, Italy; ^2^ Department of Maternal and Child Health, ASL-CN1 Child Neuropsychiatry Cuneo-Mondovì, Mondovì, Italy; ^3^ Department of Psychology, IUSTO - Salesian University Institute Torino Rebaudengo, Turin, Italy

**Keywords:** ADHD (Attention deficit and hyperactivity disorder), sex differences, gender medicine, neurodevelopmental disorders, developmental trajectories, executive functions, social functioning, impairment differences

## Abstract

**Introduction:**

This systematic review aimed to synthesize existing research on the symptomatological and behavioural differences between male and female attention-deficit/hyperactivity disorder (ADHD) in individuals aged 6–18 years. ADHD is a prevalent neurodevelopmental disorder that manifests differently across genders, potentially impacting the diagnosis, treatment, and overall management of the condition.

**Methods:**

Following the Preferred Reporting Items for Systematic reviews and Meta-Analyses 2020 (PRISMA guidelines), we conducted a comprehensive literature search and identified 67 records published between 2008 and 2024 that met our inclusion criteria. The review examined both direct sex differences—comparing female ADHD subjects to their male counterparts—and the disorder's sex-specific effects, revealing nuanced patterns of compromission.

**Results:**

Findings were organized into seven thematic areas: core symptoms, executive and attention performance, neuropsychomotor aspects, psychopathological aspects, behavioural and social aspects, substance use and academic performance. Differences between males and females with ADHD have been highlighted across several domains, including prevalence and intensity of core symptoms, cognitive functioning, and the nature of externalizing vs internalizing behaviours. Notably, variations were observed in the ways symptoms manifest, such as in aggression and emotional regulation. Furthermore, the review highlighted how ADHD's impact is influenced by the subject's sex, specifically affecting neuropsychomotor development, social interactions, and self-esteem. Age-related differences concerning the evolution of symptoms and cognitive functions were also explored, shedding light on how developmental trajectories may differ between sexes.

**Conclusion:**

A comprehensive understanding of sex specificity in relation to ADHD is critical for informing effective diagnosis and treatment strategies. This review underscores the need for further research to elucidate these differences, ultimately contributing to more tailored and sex-sensitive approaches in ADHD management.

**Systematic Review Registration:**

https://doi.org/10.37766/inplasy2025.4.0093, identifier INPLASY202540093.

## Introduction

Neurodevelopmental disorders (NDDs) encompass a range of conditions that arise during brain development, typically involving difficulties in key areas such as cognitive function, communication, motor skills, and social interaction. Notable examples of NDDs include autism spectrum disorder (ASD), attention-deficit/hyperactivity disorder (ADHD), and specific learning disorders (SLD). The causes of these disorders are multifaceted, stemming from a combination of genetic, epigenetic, and environmental factors that disrupt the intricate processes of brain development during critical periods interfere with the processes of brain development during crucial periods (1, 2).

Literature consistently demonstrates that the neurobiological development of males and females follows distinct trajectories. These sex-specific developmental pathways are particularly evident in the maturation of the frontal lobes, a brain region critical for executive function and behavioural control. The timing of frontal lobe maturation varies between sexes (3, 4). These sex-based differences in brain development may contribute to the observed variations in the prevalence and clinical presentation of neurodevelopmental disorders, specifically ADHD, in males and females (4).

According to the latest edition of the Diagnostic and Statistical Manual of Mental Disorders (DSM-5-TR), Attention-Deficit/Hyperactivity Disorder (ADHD) is *a neurodevelopmental disorder characterized by persistent patterns of inattention and/or hyperactivity-impulsivity that significantly interfere with functioning or development.* The DSM-5-TR criteria for ADHD diagnosis require the onset of symptoms before the age of 12 and their presence in multiple settings (home, school, work, social situations) for at least six months (5).

*Inattention* refers to difficulties in sustaining focus, following through on instructions or finishing work or chores, planning and organization, that are *not attributable to defiance or lack of comprehension*; *Hyperactivity* refers to *excess motor activity, talkativeness and fidgeting when not appropriate; Impulsivity* refers to *hasty actions*, done *without forethought*, that may cause harm to the individual (5).

When comparing the last three editions of the diagnostic manuals—DSM-IV-TR, DSM-5, and DSM-5-TR—a change in the description of ADHD is evident regarding its behavioural variants. The DSM-IV-TR categorized ADHD into three subtypes: Predominantly Inattentive, Predominantly Hyperactive-Impulsive, and Combined. However, the DSM-5 and DSM-5-TR have shifted to using “presentations” instead of “subtypes” to acknowledge the dynamic nature of ADHD symptoms, recognizing that individuals may experience varying combinations of symptoms over time. This shift reflects the understanding that ADHD presentations can evolve across the lifespan. Scientific studies on the subject have shown that children with a predominantly inattentive variant, at a given moment of their physiological and neuropsychological development, may evolve into different variants over time (5–9).

Furthermore, the age criteria for diagnosis have evolved across DSM editions. The DSM-IV-TR required some symptoms to appear before age 7, the DSM-5 requires several symptoms to appear before age 12, the DSM-5-TR requires some symptoms before age 12 (5–7).

The International Classification of Diseases (ICD) is another diagnostic classification system. The ICD-10, which categorized ADHD as "*hyperkinetic disorder*," required symptom onset before age 6 for diagnosis (10). In contrast, the current ICD-11 recognizes that ADHD can sometimes present later in childhood, although it emphasizes the need for careful clinical judgment when diagnosing individuals with symptom onset after age 12 (11). Both the ICD and DSM classifications have shown a shift towards placing less emphasis on the specific age of symptom onset in recent years.

The prevalence of ADHD in children aged 3–12 years is estimated to be approximately 7.6% (95% CI: 6.1%–9.4%), although this figure can vary significantly depending on the specific diagnostic criteria employed (DSM-IV, DSM-IV-TR, DSM-III, DSM-V, ICD-10), with reported prevalence rates ranging from 4.4%–11.3%. In adolescents aged 12–18 years, the prevalence of ADHD is estimated to be around 5.6%, again with variability based on diagnostic approaches (12).

Ramtekkar et al. (13) examined the prevalence of different ADHD presentations—predominantly inattentive (IN), combined (C), or predominantly hyperactive-impulsive (HI), in a large population sample *(n* *=* 3,040), considering both age and sex. In children under 12 years, the inattentive presentation was most common (33.2%), followed by the combined (31.1%) and hyperactive-impulsive presentations (23.1%). This pattern was similar in the adolescent sample *(n* *=* 151, 12–18 years). However, in clinical settings, the combined presentation is typically the most frequent.

Research findings on sex differences in ADHD subtype prevalence are inconsistent. Some studies suggest a higher prevalence of the inattentive subtype in females (14, 15), while others find no significant sex differences (15–17). Owens and colleagues suggest that this reported sex difference, like the one in ADHD general prevalence, may be more apparent in clinical samples, opposed to community ones (15).

ADHD frequently co-occurs with other mental health conditions. A study of 2,861 Italian children and adolescents with ADHD found that 66% experienced at least one psychiatric comorbidity, with the prevalence of comorbidities ranging from 40%–80% across different studies (18, 19). Commonly reported comorbidities included learning disorders, sleep disorders, oppositional defiant disorder, and anxiety disorders. The prevalence of depression in this particular study (5%) was notably lower than reported in other studies (20%–30% in individuals with ADHD), potentially due to the specific characteristics of the study sample (19).

Other disorders frequently observed alongside ADHD include bipolar disorder (prevalence ranging from 11%–75%), tic disorders (20%), obsessive-compulsive disorder (6%–15%), and autism spectrum disorder (59%–80%) (18, 19).

Regarding sex distribution, ADHD is more commonly diagnosed in males. Clinical studies have reported a male-to-female diagnosis ratio of approximately 4:1. However, this ratio significantly decreases to 2.4:1 when the analysis focuses on individuals exhibiting ADHD symptoms within the general population, suggesting that ADHD may be underdiagnosed in females (8, 20).

Madsen et al. (21) conducted a large-scale study involving parental reports from 51,527 children. Their findings revealed that girls exhibiting ADHD symptoms and behaviours were significantly more likely to remain undiagnosed compared to boys. This suggests that ADHD may be underdiagnosed in girls, potentially due to a lack of recognition of the disorder in females. This under-recognition may be partly explained by the fact that girls with ADHD are less likely to display the “classic” disruptive and impulsive behaviours typically associated with the disorder in boys (22).

These findings are supported by a recent review by Hinshaw et al. (23), who highlight several factors that help explain the underdiagnosis and under-representation of females with ADHD. These include clinicians' belief that the disorder was rare in girls and even rarer in women, based on the assumption that ADHD only affected children; the prevalence of inattentive symptoms in girls and the lesser manifestation of externalizing behaviours; a lower co-occurrence of behavioural disorders in girls or their later onset; clinical and diagnostic biases, such as the assumption that symptoms are the same in both males and females; the tendency of parents and teachers to underreport ADHD behaviours in girls, despite them being as pronounced as in boys; and the greater use of compensatory behaviours by girls. These misbeliefs in clinical diagnosis are evident across many research areas. In fact, until about three decades ago, research samples in behavioural and biomedical studies were predominantly male, including studies on ADHD. However, in 1994, a change in US guidelines began a process aimed at ensuring equal female representation in medical and scientific research.

This bias can be found in the DSM-IV, where the female reference sample for analysing ADHD symptoms was only 21%, leading to criteria that were more aligned with male characteristics (24). In the DSM-5, this bias was reduced: in the two clinical trials that included ADHD diagnostic tests, the male percentages were 69.7% and 63.8% (25).

This systematic review aims to investigate sex-related differences in ADHD among children and adolescents aged 6–18. The neurobiological development of males and females occurs at different times, and this difference is reflected in various characteristics that may develop at different rates, even without the presence of ADHD (16, 26). Understanding these differences is crucial for developing more targeted and effective treatment approaches.

**Figure 1:**
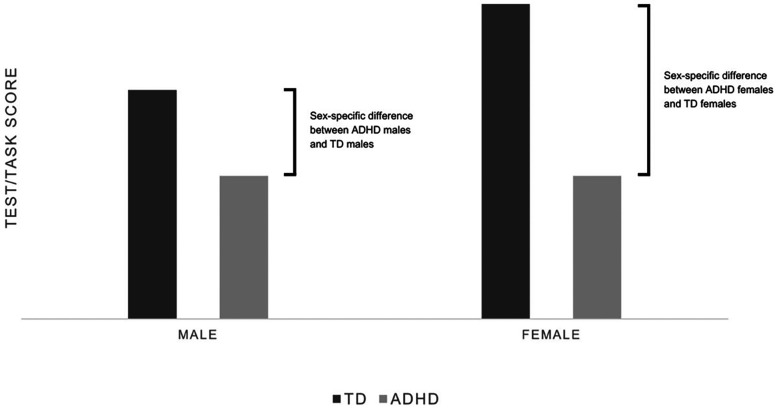
Hypothetical example of scores. No difference emerges in direct comparison between ADHD males and females. At the same time, comparing their respective differences with TD peers, the intra-sex effect of ADHD is appreciably bigger in the female sample.

The evaluation of these differences can be conducted through two comparison methods:

• The first method is a direct comparison between males and females with ADHD, for example, by comparing the results of a specific task or test. A limitation of this method is that it does not take into account the differences in neurobiological development, nor those present in the neurotypical sample.
• The second method is the comparison of the *intra-sex* or *sex-specific* effect, which can only be obtained with the presence of a control group. The sex-specific effect is obtained by comparing the results of the male ADHD sample with those of the male neurotypical (TD) sample, thus obtaining a difference (deficit). This difference is then compared with the difference between the female ADHD sample and the female TD sample (Figure 1).

## Methods

The PRISMA 2020 guidelines were followed for this review (27). The inclusion and exclusion criteria were as follows:

Included articles: those published between 2008 and march 6 2024, involving a human population aged 6–18 years, with the terms “*ADHD*” or “*attention deficit hyperactivity disorder*” or similar terms in the title or abstract; exclusively in English; and containing terms such as “*male*,” “*masculine,” “female*,” “*feminine*,” “*sexual dimorphism*,” “*sexual difference*s,” or “*gender differences*” or similar terms in the title or abstract.
Excluded articles: those investigating diagnostic tools, examining comorbidities, theses, single case studies or case reports, those not written in English, and those analysing the disorder using animal models.

The review protocol was registered on the international platform of registered systematic review and meta-analysis protocols (INPLASY) and is available in full on inplasy.com (10.37766/inplasy2025.4.0093).

Formulation of the research question was structured with the PICO scheme, specifically referring to Nishikawa-Pacher's (28) universal application of it:

• P (Problem): Population with ADHD between the age of 6 and 18
• I (Intervention): Existence of an impact of subject sex on symptoms and behaviour
• C (Comparison): Null hypothesis
• O (Outcome): Sex differences in ADHD symptoms and behaviour between males and female, aged between 6 and 18 years old

For the literature search, the following databases were selected: PubMed, PsycINFO, PsycArticles and SCOPUS. Specific search strings were created and executed within these databases:

• [male*(Title) OR boy(Title) OR masculine(Title) OR man(Title) OR men(Title) OR gender(Title) OR “Sex Characteristics”(Mesh)] AND [female*(Title) OR feminine(Title) OR girl*(Title) OR woman(Title) OR women(Title) OR gender(Title) OR “Sex Characteristics"(Mesh)] AND [“Attention Deficit Disorder with Hyperactivity"(Mesh) OR “attention deficit hyperactivity disorder"(Title) OR ADHD(Title)] AND [behavio*(Title/Abstract) OR symptom*(Title/Abstract) OR manifestation*(Title/Abstract) OR feature*(Title/Abstract) OR trait*(Title/Abstract) OR “Symptom Assessment"(Mesh)]
• (TI male* OR TI boy* OR TI masculine OR TI man OR TI men OR TI gender OR DE “Human Sex Differences”) AND (TI female OR TI feminine OR TI girl* OR TI woman OR TI women OR TI gender OR DE “Human Sex Differences”) AND (DE “Attention Deficit Disorder with Hyperactivity” OR TI “attention deficit hyperactivity disorder” OR TI ADHD) AND (TI behavio* OR AB behavio* OR TI symptom* OR AB symptom* OR TI manifestation* OR AB manifestation* OR TI feature* OR AB feature* OR TI trait* OR AB trait* OR DE “Symptoms” OR DE “Diagnosis”).
• [TITLE(male* OR boy OR masculine OR man OR men OR gender) OR INDEXTERMS(“Sex Characteristics”)] AND [TITLE(female* OR feminine OR girl* OR woman OR women OR gender) OR INDEXTERMS(”Sex Characteristics”)] AND [INDEXTERMS(”Attention Deficit Disorder with Hyperactivity”)OR TITLE(attention deficit hyperactivity disorder OR ADHD)] AND [TITLE-ABS-KEY(behavio* OR symptom* OR manifestation* OR feature* OR trait*)OR INDEXTERMS(”Symptom Assessment”)]

The initial search yielded a total of 684 records. Using Zotero®, duplicate records were removed, resulting in 458 unique records. Subsequently, three independent raters (two psychologists and one child neuropsychiatrist) screened the titles and abstracts of these records. This process led to the exclusion of 367 articles, leaving 91 articles for full-text review on which the *assessment* was conducted.

**Figure 2:**
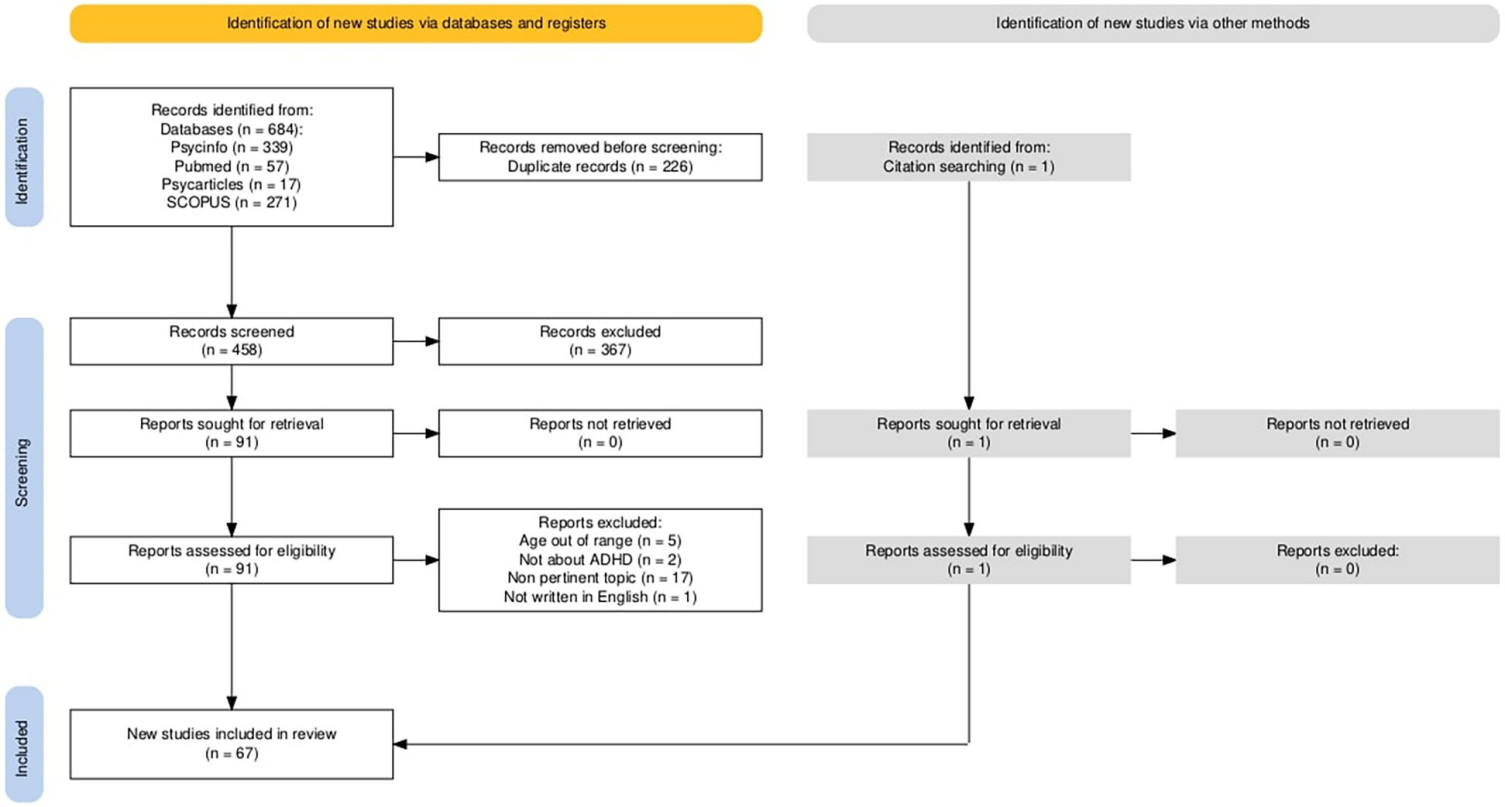
The selection process - PRISMA (Preferred Reporting Items for Systematic Reviews and Meta-Analysis).

During the assessment phase, 25 additional articles were excluded, bringing the total number of records to 66. The criteria for evaluating the articles during this phase were consistent with those used in the screening phase. Specifically, 5 articles were excluded due to the sample's age; 2 articles were removed for not focusing on ADHD; and 17 articles were excluded because their topics were not aligned with the research focus (e.g., they addressed pharmacotherapy, genetic factors, lacked exploration of sex differences, or examined the validity or reliability of diagnostic items) and 1 was excluded because the manuscript was not written in English. An additional study from another source was included, bringing the final count to 67 studies (Supplementary Tables S1, S2). A flowchart was created to visually represent this process, adhering to PRISMA 2020 guidelines and utilizing the PRISMA_flowdiagram web app (Figure 2) (29).

Bias risk assessment was conducted using checklists from the Critical Appraisal Skills Programme (CASP®) for all articles excepts for narrative reviews, since CASP does not provide one suitable. In those cases, the latest available Scale for the quality Assessment of Narrative Review Articles (SANRA) was used (30, 31, 88).

## Sex differences in ADHD core symptoms: hyperactivity, impulsivity, and inattention

Within the context of ADHD, it is crucial to consider sex differences in the manifestation of core symptoms, which present differently in males and females. This systematic review examines 13 studies focusing on inattention and self-regulation, encompassing the ability to control impulsivity, emotions, motor and verbal activity, and delay gratification.

Regarding hyperactive and inattentive symptoms**,** two studies on non-clinical population analysed sex differences using parental and teacher reports. Parental reports did not reveal significant differences, however teachers reported higher levels of hyperactivity in boys compared to girls (32, 33) Studies conducted on clinical samples do not present concordant results for inattention or hyperactivity. About inattention two of them find no significant differences and the other three find results in opposition: two finds more inattention in boys, one more inattention in girls; about hyperactivity two find no difference, three find more hyperactivity in boys (34–38). A recent meta-analysis and two studies on clinical population, utilizing data from both parental and teacher reports, demonstrated that boys with ADHD exhibit significantly higher levels of hyperactivity-impulsivity compared to girls. Furthermore, teacher reports consistently indicated higher levels of inattention in boys with ADHD (39).

Various explanations have been proposed to account for these observed sex differences. One possibility is that teachers may under-recognize inattention in girls due to the reduced presence of overtly disruptive behaviours typically associated with boys with ADHD (such as restlessness, interference during lessons, and oppositionality) which could lead to a reduced perception of inattention-related symptoms (4, 39). Additionally, Hinshaw et al. (23) suggest that girls with ADHD may exhibit hyperactivity through alternative means, such as increased verbalization, rather than the more overt disruptive behaviours commonly observed in boys.

Regarding impulsivity, a study carried out on 156 adolescents with ADHD (91 males, 65 females) found significantly higher scores in the *attention and motor impulsivity* subscales in girls compared to boys, while no significant differences were observed in non-planning impulsivity. The multiple regression analysis confirmed a significant correlation between sex and total impulsivity scores (attention and motor impulsivity subscales). The authors suggest that the sex difference observed may, in part, be explained by differences in treatment: males with high impulsivity tend to receive treatment earlier than females (40). A meta-analysis of Continuous Performance Test (CPT) studies, done by Hasson and Fine in 2012 analysed Commission errors (responses to a non-target stimulus, related to impulsivity) and Omission errors (failures in responding to a target stimulus, related to inattention). Males with ADHD made more commission errors than female with ADHD, while no difference was noted in omission error. Furthermore, a sex-specific difference was noted: compromission related to ADHD on commission errors was worse for males than female (41).

The different symptomatic trajectories have been investigated in two longitudinal studies conducted on community samples (849 and 1,571 children and adolescents) each lasting 8 years. The results indicate that females exhibit a peak in impulsivity symptoms during early adolescence, while males show a higher number of symptoms during childhood (42, 43).

According to Murray's findings, female trajectories of hyperactivity/impulsivity symptoms are divided into three classes: *low stable, high stable, and concave*. In the concave trajectory, symptoms began to increase during early adolescence (42). Also, within the female sample, for hyperactivity/impulsivity symptoms, different findings were recorded in the study done by Eng et at., which indicated a decrease in symptoms with increasing age. This decrease was more pronounced in individuals who presented with more severe symptoms at baseline (43).

Murray et al. (42) categorized the male population with regard to hyperactive/impulsive symptoms into three groups: *low stable, high stable, and high increasing*, with adolescence identified as the stage where symptoms tend to rise. However, Eng et al. found a decrease in symptoms over time for the male group, with a more significant decline observed in those who had more severe symptoms at baseline. They also noted a sex difference in the trajectory: males (who exhibited more symptoms) experienced a more substantial decrease compared to females (43).

Attention symptoms also seemed to develop differently across sexes. In this case, males were categorized into stable low and stable high, while females were divided into stable low, stable moderate, and high decreasing. In the high decreasing group, symptom levels started high but gradually decreased over time (42). Eng et al. (43) observed different patterns in symptom trajectories, with some individuals experiencing an increase in inattention while others showed a decrease. They also found a sex difference, though contrary to Murray's findings. In males that showed a reduction in inattention, those with more severe baseline symptoms experienced a more significant reduction. These results align with the high decreasing group in Murray's study, but they apply to males rather than females.

These findings suggest that adolescence should be considered a critical period for the development or onset of symptoms, which has important implications for current diagnostic criteria. The existing criteria require symptoms to appear before the age of 12, potentially excluding girls with significant symptoms and leading to underdiagnosis (5, 42, 43).

Lastly, conflicting results have emerged regarding the ability to delay gratification. The study by Mphahlele et al. (44) found no sex difference but emphasized that ADHD itself plays a central role. Regardless of sex, individuals with ADHD tend to favour immediate rewards. On the other hand, the systematic review by Carucci et al. (26) identified a reduced ability to delay gratification in females with ADHD.

## Executive functions and attention performance

The term “*Executive Functions*” (EF) encompasses a constellation of higher-order cognitive processes, including inhibition, working memory, cognitive flexibility, and planning. Extensive research has demonstrated a strong association between ADHD and deficits in EF though these difficulties manifest differently depending on sex (15, 45). While O'Brien et al. (16) found no significant differences in EF performance across ADHD subtypes, their study, involving 56 children with ADHD (26 girls) and 90 typically developing (TD) children (42 girls aged 8–13 years), revealed distinct sex-specific patterns of EF deficits in children with ADHD with significant deficits in all four EF components compared to the control sample.

The systematic review of the literature encompassing nine studies identified significant effects of sex on *planning performance and conscious response inhibition* in individuals diagnosed with ADHD (4, 15, 16, 22, 23, 26, 39, 46, 47). While no significant sex differences were observed in planning abilities within the ADHD cohort, comparisons to sex-matchet TD peers revealed a more pronounced impairment in planning abilities among females with ADHD compared to males with ADHD (4, 15, 16, 26, 39).

Boys with ADHD tend to perform worse and show a larger sex-specific gap in *conscious response inhibition* (the ability to override an automatic or impulsive reaction in favour of a more appropriate response) compared to girls with ADHD (4, 15, 16, 20, 22, 23, 26, 39, 46, 47).

Further evidence of response inhibition difficulties was revealed in a longitudinal study on 353 individuals with ADHD (aged 8–17, including 104 girls) and 241 TD controls. In this study, researchers employed a Go/No-Go (GNG) task with two levels of complexity, the more difficult version requiring increased working memory. The findings indicated that both boys and girls with ADHD had poorer response inhibition than their TD counterparts, but the specific patterns of impairment varied. Boys with ADHD showed more significant deficits and higher variability in both simple and *complex GNG* tasks. Girls with ADHD, on the other hand, had impaired response inhibition only in the complex GNG task and exhibited greater variability in both task types.

Age-related differences were also noted: for tasks with a low working memory demand, the sex-specific gap in cognitive control grew larger with age in girls with ADHD, while it shrank in boys with ADHD. Conversely, in the more complex GNG task, the sex-specific gap in cognitive control lessened with age for both boys and girls with ADHD. These sex differences may be linked to distinct developmental patterns of the frontal lobes: males might experience a delayed maturation of fronto-cerebral regions, which could account for the narrowing of the sex-specific gap during adolescence (47).

In terms of *cognitive flexibility*, Skogli's studies did not identify significant differences between boys and girls with ADHD. However, this conclusion is contested by a meta-analysis conducted by Loyer-Carboneau et al., which reviewed 11 clinical studies and found that boys with ADHD faced greater challenges with cognitive flexibility (39, 45, 48).

The two studies on *response preparation* yielded differing results: a study by O'Brien et al. [56 children (26 girls) with ADHD, and 90 controls (42 girls), aged 8–13 years] found similar performance between boys and girls. However, the study by Mahone et al. [60 children with ADHD (24 girls), and 60 typically developing children (29 girls), aged 8–12 years] found that girls with ADHD had significantly poorer response preparation compared to the boys. This discrepancy may be attributed to the specific task used in Mahone et al.'s study, the Visually Guided Saccades task (16, 49).

*Visuospatial reasoning* is addressed in two systematic reviews, both of which report that females with ADHD perform worse than males in this domain (26, 37).

Regarding attention difficulties in its various subcomponents (sustained attention, divided attention, and auditory attention), two articles were examined. In the study by Günther et al. (50), which compared children with ADHD-C to typically developing children (aged 8–14, with 48% female participants), the results showed sex-related differences, but no distinction in how ADHD impacts attentional performance. This suggests that the attentional systems of both boys and girls are equally affected by the disorder.

In the area of auditory attention, a study involving 220 children aged 7–12 years (50% female) explored potential sex differences. The findings revealed that females with ADHD had more impaired performance in auditory attention and auditory sensitivity compared to their male counterparts. On the other hand, the analysis of scores on tasks related to visual and auditory impulsivity showed that boys performed worse (51).

Finally, when considering the impact of emotions on executive functioning, executive functions (EF) are categorized into “*hot EF*” (which involve high emotional salience) and “*cold EF*” (which involve low emotional salience). A longitudinal study of 122 children (75 with ADHD and 47 without, with 44% females), aged 9–16 years, found no significant differences in cold EF between boys and girls with ADHD, a result consistent with other studies. In terms of hot EF, there were no performance differences between boys and girls with ADHD, which contrasts with the typically developing (TD) population, where boys tend to perform better than girls. Time appeared to be an influencing factor: at the baseline, girls with ADHD performed better than girls in the TD group. However, after approximately two years, the performance of the ADHD group declined, while the TD girls showed improvement. The authors suggest that girls with ADHD may initially adopt a strategy similar to that of TD boys, but the use of this strategy could diminish as they develop. These findings imply that the expression of ADHD symptoms in females follows different developmental trajectories compared to males (45).

## Neuropsychomotor aspects

In recent years, there has been a growing interest in exploring the neuropsychomotor aspects associated with ADHD, with a focus on the interaction between neurological, behavioural, and motor processes. Children with ADHD generally show significantly poorer performance in motor skills (both fine and gross) compared to typically developing (TD) children (52). The review of the literature identifies six studies that address this topic. When comparing motor skills between males and females with ADHD, the data suggest comparable performances for both fine and gross motor skills (52, 53). However, when examining sex-specific differences in comparison to the control group, Fliers et al. observed a more pronounced sex-specific difference in the female ADHD group. The gap between females with ADHD and TD females was greater than the difference observed between males with and without ADHD, in both fine and gross motor tasks (52).

ADHD also appears to have sex-specific effects in motor overflow (involuntary movements accompanying voluntary actions). In a study of 146 children (aged 8–13, with 68 females), it was found that ADHD had a significantly negative impact on the performance of females. This effect was not observed in males with ADHD compared to TD males (16).

There may also be a sex-related influence on the development of motor overflow and dysrhythmia (errors in timing or rhythm during controlled movements). A longitudinal study of 268 children (132 with ADHD, 32% females, aged 7–15 years) found that females with ADHD showed significant improvements in both motor overflow and dysrhythmia, whereas males with ADHD showed only slight improvements over time (46). In contrast, the review by Carucci et al. (26) observed improvements in overflow performance, but not in dysrhythmia, even in the male sample. These improvements were noted during adolescence, and the discrepancy in findings might be due to the older age group in Carucci's study. Regarding *mirror overflow* (involuntary movements that mirror voluntary ones), males with ADHD performed significantly worse than their TD counterparts, while this difference was not found in females (26).

Additionally, sex differences in children with ADHD can be found in the development of oculomotor control, as noted in a case-control study of 60 children with ADHD (24 females, aged 8–12). The study found that females with ADHD exhibited a slower oculomotor response latency compared to males with ADHD (49).

ADHD could also be related to persistence or re-expression of primitive reflexes, to test this hypothesis bob et al. (54) measured Asymmetric Tonic Neck Reflex (ATNR) and Symmetric Tonic Neck Reflex (STNR) in 80 children with ADHD and 60 TD children (50% females, 6–11 years old). Results show that ADHD symptom strongly associate with ATNR in girls and STNR in boys, suggesting that these reflexes were not sufficiently inhibited during development. These data support the existence of different neurological pathways in girls and boys with ADHD. Further research could explore sex-based neurorehabilitation ADHD therapies, focused on integration of primitive reflexes (54).

## Psychopathological aspects

ADHD is a neurobehavioural condition that manifests differently based on sex, affecting the way symptoms are experienced and the types of behaviours exhibited. Recent studies suggest that boys are more likely to display externalizing behaviours, such as hyperactivity and aggression, while girls tend to show internalizing symptoms, like anxiety and depression. To provide a thorough analysis, we have chosen to examine these two broad categories separately.

### Internalizing symptoms

The systematic review of the literature identified 15 articles examining sex differences in internalizing symptoms among individuals with ADHD. Females with ADHD were found to experience more internalizing symptoms compared to males, such as anxiety (48, 55), depression (56, 57), or both (4, 15, 37, 58, 59). A single study, done by Mayes et al. (60) specifically highlighted a somatic symptom, primarily present in females, related to frequent stomach-aches. Another study, which assessed Quality of life in children with ADHD reported significantly lower self-reported quality of life in girls (61).

The general statement that “*females experience more internalizing symptoms*” holds true, but a closer look at the subtypes of ADHD suggests a bidirectional interaction between sex and ADHD subtype. For example, research has shown that boys with ADHD-C are more likely to have mood disorders compared to girls with the same subtype (4).

The development of internalizing symptoms, particularly depressive symptoms, also varies by sex. In a longitudinal study by Eng et al., boys presented higher depressive symptoms at baseline, but these symptoms tended to decrease over time (43). In contrast, females with ADHD had lower depressive symptoms at baseline, which increases as they entered adolescence (15, 43). This pattern is consistent with findings on suicide attempts, which rise in girls during adolescence but remain stable in boys with ADHD (43). Furthermore, females with ADHD generally report more suicidal ideation than males with the disorder (37).

There could also be a sex difference in the relationship between evolving ADHD and depressive symptoms. A 2-year follow-up study (75 ADHD, 48% females; 27 TD, 38% females; 9–16 years old), based on self- and parent-rated depression symptoms, reports that a reduction in hyperactivity/impulsivity was associated with reduced self-rated depressive symptoms in boys, and an increase in girls. One of the proposed explanation is that impulsive behaviours could act as a maladaptive emotional regulators, and that in girls the apparent reduction of hyperactive/impulsive symptoms could be just masking, which could cause an increase in internalizing symptom. Another unexpected result was a modest increase of self-rated depressive symptoms associated with lightening inattentive symptoms; Authors propose that having less inattention could leave children with more opportunity to delve into their thoughts and feeling, resulting in more depressive symptoms (57).

A study exploring the potential links between ADHD and non-suicidal self-injury in a sample of hospitalized adolescents found a higher occurrence of this behaviour in females with ADHD compared to males (62). However, this finding may be explained by the higher rates of self-injury in females, regardless of ADHD, as indicated by a study by Ward and Curran (63). This research looked at ADHD symptoms in a sample of individuals with a history of self-injury (124 participants, 78% female, aged 13–17). It found a greater likelihood (10.1:1) of having high ADHD scores compared to the general population, but no sex effect was found to mediate the relationship between self-injury and ADHD.

ADHD also has a significant and negative effect on self-esteem (64).

Four articles (one study and three reviews) report lower self-esteem in females with ADHD compared to males (26, 37, 59, 65), with the reviews also noting poorer coping skills in females (26, 37, 59). A study by Elkins et al. (64) examined differences based on subtypes and found a greater intra-sex difference in self-esteem in girls with ADHD-I and ADHD-HI compared to boys with the same subtypes.

Two cross-sectional studies, one on individuals with ADHD (93 participants, 34% female, aged 8–12) and the other focusing solely on ADHD-I (188 participants, 44% female, aged 7–11), found a sex effect in the relationship between internalizing symptoms and peer difficulties: in females with ADHD, internalizing symptoms were more strongly linked to lower social desirability compared to males (55, 66).

### Externalizing symptoms

ADHD generally leads to an increase in externalizing behaviours, irrespective of sex (15). The review of the literature revealed that 11 articles focused on externalizing symptoms. When comparing males and females with ADHD, it appears that males tend to display more externalizing behaviours (15, 26, 37, 48, 56, 60, 67, 68). However, when comparing the ADHD group with the neurotypical group (ADHD males vs. TD males or ADHD females vs. TD females), the increase in externalizing behaviours associated with ADHD seems to be similar across sexes (15).

A longitudinal community study (duration: 4 years; 50% females, aged 7–14, 3,893 participants) identified differences in factors that predict externalizing behaviours. Specifically, stressful life events were found to predict externalizing symptoms, but only in males. This might be due to females being more likely to react to stressful events with internalizing behaviours (67).

Another longitudinal community study (duration: 1 year; 46% females, aged 5–13, 147 participants) explored the relationship between ADHD symptoms, sex, and rule-breaking behaviour in school. The study found a significant relationship between ADHD and rule-breaking, but only in males. This may be explained by the higher prevalence of hyperactive-impulsive symptoms in males (69). Another possible explanation is the different ways in which symptoms manifest: males with ADHD often exhibit more *overt* and aggressive behaviours, while females with ADHD tend to show more covert and relational behaviours (4, 70).

## Behavioural and social aspects

ADHD is a neurodevelopmental condition that not only affects attention and impulse control but also has a major impact on individuals' behaviour and social interactions. Both males and females with ADHD face significant social difficulties compared to those with typical development. The review of the literature identifies 11 studies addressing these issues.

Concerning *problems with peers*, 5 studies examine the topic and present conflicting findings.

One case-control study by Ragnarsdottir et al. (17) was conducted with children with ADHD and TD children (592 ADHD children aged 5–10 and 215 TD children aged 6–10). The study aimed to explore age and sex differences in social difficulties and prosocial behaviour. Results showed that children with ADHD had notably more peer problems and exhibited less prosocial behaviour compared to the control group. Sex related differences within the ADHD group were also found, with girls with ADHD experiencing more peer difficulties than boys with ADHD. However, this sex difference was statistically significant only in parent reports. Teachers noted significantly lower prosocial behaviour in younger girls with ADHD compared to older girls. An additional finding showed an interaction between age and sex in peer problems: older girls with ADHD had peer issues similar to younger girls, while older boys had fewer peer problems than their younger counterparts.

These results are somewhat challenged by two studies, one focusing on teacher assessments and the other on parent assessments, which report more peer difficulties in males with ADHD or TD males showing high levels of ADHD symptoms (56, 71).

Additional findings come from a study by Elkins et al. (998 participants, 520 females, aged 11), which examines sex-specific effects of ADHD on experiences of bullying and social desirability. The study register that ADHD has a greater impact on females compared to males, specifically in terms of bullying, which grows more in females as effect of ADHD, and social desirability, which decreases in females (64). Furthermore, when the sample was analysed by DSM-IV subtypes, the study revealed that the sex-specific effects of ADHD on bullying and social desirability are particularly detrimental in females with ADHD-I, while in the ADHD-C group, the negative sex-specific effects are more significant in males (64). Mikami and Lorenzi (125 participants, 33% females, 6–10 years old) don't find the same sex-specific effect of ADHD, which in their sample compromises peer functioning similarly in boys and girls. This study also analysed sex-specific effect of conduct problems, registering a significantly greater effect on peer functioning in females (72).

The impact of subtypes has also been noted in a cross-sectional study conducted on a community sample of 1,775 Qatari adolescents (150 ADHD, 38% females; 1,625 TD, 61% females). Based on teacher's observation, girls with ADHD-I had significantly more social difficulties than boys with the same subtype, while no significant differences were noted for the other subtypes (73).

Another factor that may contribute to peer problems is the reduced ability to identify emotions through facial expressions (Facial Emotion Recognition, FER). This ability tends to be impaired in individuals with ADHD compared to those with typical development. However, a study by Dede and White (87 participants, aged 6–10, 56% female) did not find any significant sex-related differences (74).

Another important consideration when examining peer issues is how aggression is expressed, which appears to differ by sex: females with ADHD tend to display less physical aggression than males with ADHD but exhibit more verbal aggression compared to TD females (37, 59). Aggression can be classified as reactive (RA), which is a *defensive response to threat or provocation*, or proactive aggression (PA), which is an *instrumental, goal-directed behaviour*. The relationship between ADHD, aggression and gender could be different based on the type of aggression, as presented by a study of Vida et al., which assessed levels of PA and RA in ADHD adolescents and TD controls (391 ADHD, 391 TD; 8% females, 11–17 years old) using a self-rated questionnaire. Results show that boys with ADHD exhibit more PA behaviour than girls, while girls with ADHD exhibit more RA than boys. This last result contradicts previous research that find less externalizing problems and RA in girls. This difference could be explained by the source of information used, since other studies use teacher reports to assess aggressivity. It is known than girls with ADHD tend to show more covert aggression, which could be less detectable by teachers and possibly better represented in self-reports (4, 70, 75).

The findings regarding prosocial behaviours are not uniform, too: two studies report that females with ADHD or high ADHD symptoms show more prosocial behaviours compared to males (71, 76), while one study observes fewer prosocial behaviours in females, but only in teacher evaluations (17).

Two studies focus on social skills and yield consistent results. Both identify a primary effect of sex and an interaction between sex and age. In Ragnarsdottir et al.'s study, the male ADHD group demonstrated lower levels of social, communication, and recreational skills (such as the ability and frequency to plan and organize recreational activities) compared to the female ADHD group. Additionally, a longitudinal study by Mahendiran et al. showed different trajectories for social skills based on sex. The female ADHD group exhibited greater improvement than the male group, though no significant difference was found in communication and recreational skills, where both groups improved similarly (17, 77).

Finally, a study of 334 children (52% male, aged 8–10) explored the relationship between ADHD and best friend conflicts. Sex differences were found about which ADHD symptoms were most linked to conflicts: in the male group, hyperactive symptoms were the primary contributors to conflicts with a best friend, while in the female group, inattentive symptoms were more closely associated with such conflicts. Aggression and emotional and behavioural instability were factors that mediated these associations for both sexes (78).

## Sex differences in alcohol, tobacco, and marijuana use

The systematic literature review identified five studies that focus on sex differences in the use of psychoactive substances among individuals with ADHD.

The findings suggest that children and adolescents with ADHD are more prone to substance use than their typically developing peers (59, 79–81). Sex differences have been observed regarding tobacco use, with females with ADHD being more likely to smoke compared to males (22, 80). However, this was not supported by a longitudinal study by Lee et al. (82), which was conducted on a sample of young people in Singapore (*n* = 9,719; 54% female). The authors proposed that this difference might be specific to the Caucasian population. To better understand the relationship between childhood ADHD and tobacco and marijuana use in adolescence, Elkins et al. conducted a longitudinal study on monozygotic twins (*n* *=* 2,164; 52% female, followed from aged 11–17). The study showed that ADHD directly influences tobacco and marijuana use in females, while in males, relational difficulties with peers were the primary mediating factor, particularly regarding marijuana use. Depression and anxiety did not act as significant mediators. Another notable finding concerns self-medication: females were found to be more likely than males to use nicotine as a way to cope with attention difficulties (79).

Finally, a second study by Elkins, conducted with a sample of 3,762 participants (52% female, aged 11–17), explored how age and ADHD influence alcohol and marijuana use. The results revealed a sex difference in the relationship between hyperactivity/impulsivity and substance use, with the relationship being stronger in females (83).

Norén Selinus et al. (81), explored the relationship between substance use and ADHD symptoms in a follow up community study conducted on 4,635 children (51% females, 15 years old), finding that girls with high ADHD symptomatology had a greater risk of drug abuse compared to boys with same symptoms level (81).

The combination of peer relationship difficulties, ongoing ADHD symptoms, and early substance use can negatively impact development, indirectly contributing to the issues with tobacco and marijuana use seen in individuals with childhood ADHD (79).

## Academic performance

A review of six studies on academic performance reveals sex differences. In terms of mathematical skills, a two-year longitudinal study involving 958 children (49% girls, average age in baseline: 7.3 years) in a non-clinical group examined how sex differences in inattentiveness and hyperactivity/impulsivity traits impacted math performance. The results showed a negative correlation between inattention and math performance and a positive correlation between hyperactivity and math performance. Additionally, sex differences on the effect of age were observed: both boys and girls showed a negative correlation between inattention and math performance, but this relationship weakened over time in boys, while it remained stable in girls. As a result, the performance gap between boys with low and high inattention decreased, while it remained the same for girls (84).

Silva et al. (85) examined sex differences in numeracy in a sample of 21.270 children (6.819 with ADHD; 20% females; 7–11 years old), reporting that girls with ADHD were more impaired than boys.

A study by Sturm et al. (86) examined factors influencing mathematical calculation skills in a sample of 281 children (30% females, aged 8–15) diagnosed with ADHD. The study found that auditory-verbal memory and processing speed could be important predictors of math performance. Children who performed better on the Wechsler Intelligence Scale for Children Fourth Edition (WISC-IV) working memory task (*letter-number sequencing*) also performed better in math tasks. A three-way interaction between sex, anxiety (specifically anxious perfectionism), and processing speed was identified in relation to math performance. The impact of anxious perfectionism and processing speed on math performance was mediated by sex. In boys with low levels of anxious perfectionism, slower processing speed was a strong predictor of poorer math performance compared to girls. However, no significant sex differences were found at moderate or high levels of anxious perfectionism. These findings should be interpreted with caution due to an unbalanced sample and the absence of a control group.

When it comes to language skills, a systematic review found that girls with ADHD-C performed worse than boys with ADHD-C in verbal fluency tasks. In the inattentive subtype, however, boys performed worse. The authors suggested that these findings could indicate greater deficits in individuals with the rarer subtype for their sex. Furthermore, girls with ADHD, regardless of subtype, appeared to have weaker *vocabulary skills* (37).

Silvia et al. (85) also examined writing difficulties in their samples, measuring more impaired performances related to ADHD in boys.

Text comprehension ability also differs between boys and girls. An observational study of 131 children with ADHD (aged 8–11, 50% girls) explored how sex, inattention, hyperactivity, and impulsivity influenced reading comprehension. The study found a strong link between inattention and difficulties in text comprehension, reading fluency, and overall reading ability, regardless of sex, highlighting the crucial role of attention in reading skills. Sex differences were observed, with girls with ADHD performing better in all aspects of reading than boys. A two-way interaction between sex and inattention was also found: boys with inattentive behaviours performed worse on text comprehension than girls with similar levels of inattention. This interaction did not occur between sex and impulsivity or hyperactivity. One explanation could be that girls develop language skills earlier, which may help compensate for inattention when completing reading comprehension tasks (87). Additionally, an effect mediated by sex was observed regarding externalizing problems and text comprehension. In a study with 187 children with ADHD (72 girls, aged 7–11), girls with high levels of externalizing problems demonstrated significantly worse text comprehension, although with a small effect size, compared to girls without externalizing problems. This association was not found in boys (70).

When considering overall academic performance, girls with ADHD-I seem to be particularly affected. In a randomized controlled trial (RCT) study of 998 participants (520 girls, aged 11), the sex-specific gap in school motivation, expectations, and academic achievement was significantly larger in girls than in boys with ADHD-I (64).

## Conclusions

This review highlights significant sex differences in ADHD presentation, which appears to follow different trajectories likely due to differences in the maturation of brain networks, particularly the frontal lobes. Males are thought to experience a delayed maturation of these regions, which could explain the macro differences observed in childhood, as well as a more noticeable improvement during adolescence compared to females (26, 47).

One of the possible explanations of the higher ADHD prevalence in males may be the “*female protective effect*” theory, which propose that females require greater exposure to genetic and environmental risk factors to develop diagnosable symptoms. The observation of a higher prevalence of *causal factors* in females with ADHD may also indicate that this group consists primarily of individuals with more severe symptoms (23, 34). This may be due to a higher diagnostic threshold for females, potentially leading to underdiagnosis in girls with significant functional impairments (and corresponding *causal factors*) relative to the female typically developed population.

Recent research supports the need for sex-specific diagnostic thresholds. This would allow for the diagnosis of females who, despite experiencing impairments, cannot currently be diagnosed. The results of a recent study support this position: a sex-specific threshold for females requiring 4 symptoms instead of 6 would help identify girls with significant functional impairments. Results also support current 6 symptom threshold for males (34).

An alternative explanation for the higher prevalence of ADHD in males may be attributed to methodological artifacts. Arnett et al. explored sex differences in symptom counts, assessing whether the higher diagnosis rate reflects true etiological differences or is merely the result of methodological artifacts, including *selection bias, measurement invariance, and missing symptoms*. Even after adjusting for these potential artifacts, the study found that males still presented with a higher number and a variance of symptoms compared to females. This suggests that biological differences may contribute to the higher prevalence of ADHD in males, particularly at the severe end of the spectrum (20).

However, as noted by the authors, this study should be considered exclusively as an explanation for the symptomatic differences observed, rather than the disparity in diagnosis rates.

We deem crucial to assess the findings of Arnett et al. alongside those of Babinski et al. (34), that suggests that an individual's impairment is not solely determined by the number of symptoms. The meaning of symptom count could vary depending on the sex, thus making the use of symptom count alone to measure and compare severity and impairment potentially inaccurate.

In terms of the core symptom triad (inattention, hyperactivity, and impulsivity), male participants exhibit higher levels of all three symptoms compared to female participants (32, 33, 39). For attention deficit, the most notable differences are seen in auditory attention, where females with ADHD show greater deficits in attention and auditory acuity tests, although they perform better than males in both auditory and visual impulsivity. Regarding inattention in different environments (family vs. school), males experience greater difficulties in the school setting (39, 51).

For hyperactivity and impulsivity, male participants again show higher symptom levels across environments (39). These findings could also reflect an underestimation of the challenges faced by females due to the lower frequency of disruptive behaviours (4).

When it comes to cognitive performance, females with ADHD outperform males in areas such as reading abilities, cognitive flexibility, and conscious response inhibition (4, 15, 16, 20, 23, 26, 39). Males, however, have better performance in visuospatial reasoning and do not display impairments in planning abilities compared to their same-sex TD peers (16, 26, 37).

Research on prosocial behaviours and peer relationships in ADHD has yielded inconsistent findings regarding sex differences (17, 56, 71). However, when assessing social skills, studies consistently show that females with ADHD demonstrate greater social competence compared to males, with this advantage becoming more evident with age (17, 77).

As for psychopathological aspects research indicates that females with ADHD exhibit more internalizing symptoms, while males display more externalizing behaviours, particularly the *overt* type (16, 37, 56, 58, 67, 70).

Furthermore, females with ADHD have higher rates of nicotine use, potentially as a form of self-medication for inattention, and are at increased risk of self-harm. These issues may be exacerbated by delayed diagnosis and treatment (62, 79).

ADHD also has a more significant negative impact on females in terms of social experiences, including higher rates of bullying and lower social desirability, thus it's not surprising that studies also report lower self-esteem and increased suicidal ideation. Delayed diagnosis can significantly worsen these outcomes by increasing the likelihood of negative self-attributions and self-blame (37, 64, 65).

Since the research on sex differences in ADHD is still in its early stages, it's not unexpected the absence of definitive scientific consensus in various areas. The current body of literature is limited and faces methodological issues, such as the occasional lack of both teacher and parent evaluations in interview-based studies and the infrequent use of self-assessments by participants. The absence of multiple evaluators weakens the reliability of the findings. Lastly, almost all the articles measured and considered sex but not gender, more nuanced results could emerge if gender identity is accounted for. The inclusion of studies that use both clinical and community samples with large sample sizes strengthens the conclusions presented, but a larger data pool based on community sample would create a better representation of the population. The current literature presents great heterogeneity in measuring instruments and sources (teacher, parents or subjects), and has to be considered when interpreting results. In order to gain a clearer understanding of sex differences, we deem critical not only comparing males and females with ADHD but also exploring sex-specific differences. This allows the identification of the distinct effects of ADHD in each sex, while also distinguishing these effects from broader sex-related differences observed in TD groups. Explicit focus on this approach is, to our knowledge, absent from existing review literature and a strength of this review.

## Data Availability

The original contributions presented in the study are included in the article/Supplementary Material, further inquiries can be directed to the corresponding author.
